# Genetic identification of endoscopic biopsies after unnecessary gastrectomy: Case report and medico-legal evaluation

**DOI:** 10.1016/j.ijscr.2019.04.047

**Published:** 2019-05-07

**Authors:** Matteo Sanavio, Eugenia Carnevali, Simona Severini, Federica Tommolini, Luciana Caenazzo, Pamela Tozzo

**Affiliations:** a Department of Cardiac, Thoracic, Vascular Sciences and Public Health, University of Padova, Via Falloppio 50, 35121 Padova, Italy; b Department of Biomedical and Surgical Science, Section of Legal Medicine and Forensic Science, University of Perugia, “S. Maria” Hospital, Via Cesare Mazzieri 3, 05100 Terni, Italy; c Department of Molecular Medicine, University of Padova, Via Falloppio 50, 35121 Padova, Italy

**Keywords:** Case report, Gastrectomy, Colorectal cancer, Forensic genetics, Medical liability

## Abstract

**Introduction:**

Forensic genetic laboratories analyse samples included in paraffin to verify the genetic correspondence of histological samples, from living subjects or cadavers, in cases where there is a suspicion of contamination of samples with tissues of other patients.

**Presentation of the case:**

A case of a man subjected to a gastrectomy as a result of a histological diagnosis of gastric adenocarcinoma after endoscopic biopsies is reported. The microscopic analysis on the gastric tissue after the gastrectomy excluded the presence of cancer. Having suspected a diagnostic error, a microscopic revision of the biopsies was performed and confirmed the presence of cancer cells but led to a hypothesis that there had been contamination with foreign intestinal tissue. The genetic analysis performed on various pieces of tissue, despite the reduced amount of biological material, succeeded in identifying the presence of two incomplete genetic profiles, one of which belonged to a subject of the opposite sex.

**Discussion:**

The case raised many questions about the process of setting up histological specimens. Even though it is impossible to identify the healthcare professionals responsible for contamination, the organizational error during the management of biopsies has significantly affected the clinical case of the patient, who underwent a gastrectomy for cancer that was not present.

**Conclusion:**

This case is not simply an example of diagnostic error and related unnecessary surgery, but it has raised some doubts about patient management and it has led us to some medical-legal cause for reflection in the field of professional liability.

## Highlights

• Forensic genetic laboratories can analyse samples to verify the genetic correspondence to patient.
• Intra-hospital chain of custody of the biopsy samples is of paramount importance.
• It is essential to ensure the traceability of samples from collection throughout the work cycle.

## 1 Introduction

Nowadays, forensic genetic laboratories frequently analyse samples included in paraffin in cases of “uncertain paternity” where it is impossible to collect a biological sample directly from the subject (due to disappearance or death) [1]. Nowadays, these procedures are also performed to verify the genetic correspondence of histological samples, coming from living subjects or cadavers, in cases where there is suspicion of contamination of the samples with tissues of other patients [[2], [3], [4]]. The increasing number of these events raises many questions about the operational protocols of biological samples, in particular, during endoscopic exams.

Esophagogastroduodenoscopy (EGDS) is an upper-gastrointestinal endoscopy useful for visualizing the oropharynx, oesophagus, stomach and proximal duodenum, with real-time evaluation and interpretation. During this procedure, sampling of gastric and/or duodenal tissue is very important to perform histological, cytological and/or microbiological analyses, depending on the clinical scenario. While making the visual assessment and deciding what sampling to perform, endoscopists should always keep the clinical questions which prompted the procedure in the first place in mind [5].

When submitting samples, it is helpful to provide the pathologist, cytologist or microbiologist with details such as clinical history, specimen-specific location and appearance and the questions to be answered. An endoscopy report and photographs of the area in question can also be very helpful. Typically, biopsy forceps are positioned in the accessory channel of the endoscope, placed in the targeted area and the forceps are opened and closed to obtain a biopsy of the mucosa or to remove small polyps.

Each year, approximately 990,000 people are diagnosed with gastric cancer worldwide and about 738,000 die from this disease, making gastric cancer the 4th most common incident cancer and the 2nd most common cause of cancer death. Gastric cancer also causes one of the highest cancer burdens, as measured by disability-adjusted life years lost. Survival has been prolonged by the development of chemotherapy and molecular-targeted therapy in cases of advanced gastric cancer but surgical resection remains the most effective treatment for curable cases [6,7].

The present work has been reported in accordance with the Surgical Case Reports (SCARE) criteria [8].

## 2 Presentation of the case

The case of a 64-year-old man who performed an EGDS because of persistent symptoms (nausea, vomiting, pyrosis and weakness) is presented. During the endoscopic examination, seven gastric biopsies were obtained, originating from the antrum of the stomach for subsequent histological analysis.

This investigation revealed the presence of "adenocarcinoma in the context of high-grade (severe) glandular dysplasia islands, with pseudo-polypoid gastric mucosa and chronic inflammation". Doctors suggested the complete surgical removal of the stomach as a result.

After a short time, the patient turned to an other hospital where he underwent a gastrectomy, based on the previous histological diagnosis.

Thereafter, the stomach was completely sampled and analysed and no cancerous cells were dectected.

Due to this discrepancy, the man requested a histological review of the gastric samples obtained during the EGDS and gastrectomy, but the original diagnose in both cases was confirmed. The anatomopathologist who suspected the presence of gastro-intestinal tissues foreign to the patient, suggested a genetic analysis of all the gastric samples. At this point, the patient arrived at the Laboratory of Forensic Genetics of the University of Perugia where, after extracting DNA from the various gastric samples (formalin-fixed paraffin-embedded tissues), they were typed with Promega’s PowerPlex ESX 17 Fast and PowerPlex Y23 kits, in accordance with international protocols. A sample of saliva of the patient was also collected in order to compare the results. All formalin-fixed paraffin-embedded samples showed a mixed profile. Despite the scarcity of material examined, it was possible to repeat the analysis on each paraffin sample twice, in order to exclude any error and to demonstrate that the autosomal mixed profile was attributable, based on the height of the observed electrophoretic peaks, to a biological mixture composed of biological material from a male subject and a female subject.

Moreover, through microscopic analysis performed by a third anatomopathologist, a correlation of the different portions of gastro-intestinal tissue present in the formalin-fixed paraffin-embedded slides to the two different subjects was obtained and the presence of two different types of histological tissues was confirmed. Finally the two identified tissue fractions from the slide were analysed separately. We reached the conclusion that all the samples were composed by a neoplastic intestinal tissue containing adenocarcinoma cells (the biopsy showed dilated or slit-like tubules and lined with a columnar epithelium, branching glands or acinar structures surrounded by various degrees of desmoplasia. The diagnosis formulated was: “tubular adenocarcinoma”, following the WHO classification (2010) which corresponded to “intestinal carcinoma” according to the old Lauren classification (1965) belonging to an unknown female subject and tissue of certain gastric origin without cancer cells belonging to the patient.

## 3 Discussion

This is a case of certain medical liability where it is very difficult to identify specific errors and the professionals who made the error.

It is obvious that during the phases of sampling (EGDS), storage and/or processing, a piece of intestinal tissue coming from a female subject affected by intestinal adenocarcinoma contaminated the gastric biopsy of the male patient, who was not affected by cancer.

Being a foreign intestinal sample, the hypothesis that the insertion occurred during the sampling phase can be excluded because the endoscopes used for EGDS and for colonoscopy are different. Instead, it is possible that two biopsies, coming from separate examinations were wrongly inserted into a single container, which presented the personal data of the male subject, thus generating an error in the sampling or closing phase. However, it is even more plausible that the error occurred in the anatomo-pathology laboratory where, according to Ministerial indications, all tissue samples taken for diagnostic purposes must be stored, conserved and processed.

In particular, the extraneous female intestinal tissue may have been added to the biopsy specimen during the staging phase or during paraffin inclusion.

Unfortunately, in this case there were no preconditions raising the suspicion of a pre-surgical diagnostic error and the suspicion of contamination of biological samples arose only after *in toto* histological analysis performed on the stomach, following the gastrectomy.

The case raises many questions about the process of setting up histological specimens. Despite the correct diagnosis of a neoplasia, there was still a diagnostic error since the tissue containing the cancer cells was intestinal and not gastric. Even though it is impossible to identify the healthcare professionals responsible for the contamination, it is evident that the organizational error during the management of the biopsies significantly affected the clinical case of the patient, who underwent a gastrectomy for cancer that was not present. It is also reasonable to ask why the surgeons who removed the stomach did not ask for or perform a new EGDS, considering that the patient underwent the gastrectomy in a hospital where EGDSs are routinely performed or at least requested a second opinion on the histopathological examination of the patient’s samples. On the other hand, it is impossible in our opinion to attribute any blame to the work of the first pathologist who diagnosed the gastric adenocarcinoma instead of intestinal carcinoma. In fact, it often happens that due to dysplastic-metaplastic processes, the gastric tissue presents histo-morphological characteristics very similar to intestinal tissue, making a differential diagnosis particularly difficult.

It is not sustainable, for economic and technical reasons, to perform a genetic analysis on all bioptic samples taken for diagnostic purposes. Only if a pre-surgical review raises some diagnostic doubts about the origin of the samples, then this kind of analysis in order to verify the diagnosis before surgery is recommended. This would, first of all, allow an improvement in the quality of assistance to the patient. Secondly, it would allow savings related to compensation for damages, especially in cases where, since the error, is not easily identifiable an objective responsibility of the structure.

Careful monitoring by the endoscopist of the number of samples taken and the number of samples sent to the pathologist, represent a possible solution. For example, if the mistake had occurred in the sampling phase, too many fragments would have resulted in one sample and few (or even none) in another sample(s).

Furthermore, to limit the possibility of error in the staging phase, it is a good rule to alternate cases in different organs (gastroenteric/urologic/gynecologic, etc) with the utmost cleanliness.

Interestingly, the Italian Health Ministry has recently issued guidelines regarding Traceability, Collection, Transport, Storage and Archiving of cells and tissues for diagnostic investigations, since the conservation of these samples is a priority in order to guarantee a correct and complete diagnosis but at the same time, their custody over time is important to fulfil the need for further analysis [9]. In the same way, it is also essential to ensure the traceability of samples at the time of collection, throughout the work cycle up to archiving, to avoid errors in identification and / or loss of samples and information.

## 4 Conclusion

The case underlines two fundamental critical aspects in the management of a biopsy from the time of collection to final diagnosis.

First, it is not justifiable that a surgical department performs an operation on an organ affected by cancer, without ascertaining the histological diagnosis executed in another hospital, especially if this involves a significant impact on the patient's life.

Secondly, it is necessary to promote knowledge and respect of the guidelines on the chain of custody and management of biological samples to be initiated for diagnostic analysis.

Finally, the present case highlights the need to take care of patients in a multidisciplinary way, even among healthcare professionals working in different hospitals. In fact, it is not possible to rely only on the correct collection of biopsies, the correct histological evaluation and the correct technical execution of the gastrectomy; in this case, even if the three specialists (endoscopist, anatomo-pathologist and surgeon) probably each treated the patient in the correct way, the overall management of the patient was completely unsatisfactory.

## Conflicts of interest

Authors have no conflicts of interest to declare.

## Sources of funding

This research did not receive any specific grant from funding agencies in the public, commercial or not-for-profit sectors.

## Ethical approval

This manuscript does not refer to research involving patients, so it does not need ethical approval.

## Consent

Written informed consent was obtained from the patient for publication of this case report. A copy of the written consent is available for review by the Editor-in-Chief of this journal on request.

## Author contribution

LC and EC identified the subject and selected the case report; MS conducted the literature review and wrote the first draft of the manuscript, PT rewrote the final version of the article and approved the subject, SS and FT performed the genetic analysis. All Authors approved the final version of the manuscript.

## Registration of research studies

This is a case report, it is not referring to research involving human subjects.

## Guarantor

Dr. Pamela Tozzo.

## Provenance and peer review

Not commissioned, externally peer-reviewed.
